# The PTEN Phosphatase Controls Intestinal Epithelial Cell Polarity and Barrier Function: Role in Colorectal Cancer Progression

**DOI:** 10.1371/journal.pone.0015742

**Published:** 2010-12-23

**Authors:** Marie-Josée Langlois, Sébastien Bergeron, Gérald Bernatchez, François Boudreau, Caroline Saucier, Nathalie Perreault, Julie C. Carrier, Nathalie Rivard

**Affiliations:** 1 Canadian Institutes of Health Research Team on Digestive Epithelium, Département d'Anatomie et de Biologie Cellulaire, Faculté de Médecine et des Sciences de la Santé, Université de Sherbrooke, Sherbrooke, Québec, Canada; 2 Canadian Institutes of Health Research Team on Digestive Epithelium, Département de Médecine, Faculté de Médecine et des Sciences de la Santé, Université de Sherbrooke, Sherbrooke, Québec, Canada; Emory University, United States of America

## Abstract

**Background:**

The PTEN phosphatase acts on phosphatidylinositol 3,4,5-triphosphates resulting from phosphatidylinositol 3-kinase (PI3K) activation. PTEN expression has been shown to be decreased in colorectal cancer. Little is known however as to the specific cellular role of PTEN in human intestinal epithelial cells. The aim of this study was to investigate the role of PTEN in human colorectal cancer cells.

**Methodology/Principal Findings:**

Caco-2/15, HCT116 and CT26 cells were infected with recombinant lentiviruses expressing a shRNA specifically designed to knock-down PTEN. The impact of PTEN downregulation was analyzed on cell polarization and differentiation, intercellular junction integrity (expression of cell-cell adhesion proteins, barrier function), migration (wound assay), invasion (matrigel-coated transwells) and on tumor and metastasis formation in mice. Electron microscopy analysis showed that lentiviral infection of PTEN shRNA significantly inhibited Caco-2/15 cell polarization, functional differentiation and brush border development. A strong reduction in claudin 1, 3, 4 and 8 was also observed as well as a decrease in transepithelial resistance. Loss of PTEN expression increased the spreading, migration and invasion capacities of colorectal cancer cells *in vitro*. PTEN downregulation also increased tumor size following subcutaneous injection of colorectal cancer cells in nude mice. Finally, loss of PTEN expression in HCT116 and CT26, but not in Caco-2/15, led to an increase in their metastatic potential following tail-vein injections in mice.

**Conclusions/Significance:**

Altogether, these results indicate that PTEN controls cellular polarity, establishment of cell-cell junctions, paracellular permeability, migration and tumorigenic/metastatic potential of human colorectal cancer cells.

## Introduction

Colorectal cancer (CRC) is the second leading cause of cancer-related death in Western countries. A hallmark of colorectal cancer is loss of cellular organization. The histological grade of colorectal carcinomas is an important prognostic variable and is dependent on the degree of glandular differentiation and cellular polarity. High-grade, poorly differentiated colorectal neoplasms are usually more aggressive than their low-grade, well-differentiated counterparts [1].

In epithelial cells, cell-cell adhesion and apical-basal polarity are maintained through the formation of several intercellular adhesion systems such as tight junctions (TJs). The TJ regulates paracellular diffusion and functionally segregates the plasma membrane into two compartments, which is a requirement for full polarization of epithelial cells [2]. Neoplastic cells frequently exhibit structural and functional deficiencies in both tight and adherent junctions [3]–[5]. As reviewed by Martin and Jiang [4], the expression of many tight junction proteins is disregulated in colorectal cancers. The adherens junction protein E-cadherin is also diminished in invasive colorectal cancers [6]. Additionally, the expression pattern of hDlg and hScribble, known to control the establishment of apical-basal polarity in epithelial cells [7], markedly changes during colorectal tumor progression with down-regulation of both proteins being associated with lack of epithelial cell polarity and disorganized tissue architecture [8].

Phosphoinositides have also emerged in recent years as general determinants of both polarity and identity of several membranes. Recent data indicate that PtdIns(4,5)P2 is a key determinant of the apical surface in epithelial cells [9]. Indeed, in non-polarized MDCK cells, both PtdIns(4,5)P2 and PtdIns(3,4,5)P3 colocalize at cell-cell and cell-extracellular matrix contacts. However, during the early stages of polarization, PtdIns(4,5)P2 becomes mostly concentrated at the apical membrane of the cells whereas PtdIns(3,4,5)P3 remains localized to the basolateral membrane and excluded from the apical membrane. The lipid phosphatase PTEN localizes to the apical domain and is essential for the segregation of PtdIns(4,5)P2 to the apical surface due to its dephosphorylating action on the D3-phosphate group of PtdIns(3,4,5)P3 [10]. Furthermore, PTEN acts as a negative regulator of the Phosphatidyl Inositol 3-Kinase (PI3K)/Akt signaling pathway and has been shown to influence many processes deregulated in tumorigenesis such as proliferation, cell survival, migration and invasion [11], [12].

PTEN is encoded by the *phosphatase and tensin homolog deleted on chromosome 10* (*pten*) tumor suppressor gene, which is the second most frequently mutated gene in human cancers following *TP53* [12]. Furthermore, loss of PTEN immunostaining in colorectal cancers tissues has been associated with advanced disease, liver metastasis and poor patient survival [13]–[16], suggesting its potential protecting role against progression of human colorectal carcinogenesis. Since PTEN controls polarity in normal epithelial cells, one might speculate that the loss of this protein may be sufficient to trigger epithelial-mesenchymal transition (EMT), a critical early event in the invasion and metastasis of many types of cancer, including CRC. In previous studies, the effects of PTEN loss have primarily been measured in cancer cell lines which harbor numerous other transforming and oncogenic mutations and which have lost their epithelial phenotype [17]–[19]. Such an approach has made it difficult to determine which phenotypes are directly conferred by the loss of PTEN and for defining the stages of tumorigenesis that are specifically altered in cells with PTEN loss. In order to further characterize the link between PTEN loss, loss of polarity and colorectal cancer progression, we used the Caco-2/15 cell line derived from a relatively well-differentiated human colorectal adenocarcinoma. This clone of the parent Caco-2 cell line has been extensively characterized for its ability to undertake a full morphological and functional intestinal epithelial differentiation process which takes place spontaneously once confluence has been reached and which is completed after 15–20 days of post-confluence. More specifically, these colonic cells form apical tight and adherent junctional complexes and form a polarized monolayer after several days of post-confluence, exhibiting transepithelial electric resistance (TEER) similar to *in vivo* observations [20]–[22]. The following “de-differentiated” CRC cell lines were also analyzed: the HCT116, a microsatellite-unstable human CRC cell line and CT26, a mouse metastatic colon carcinoma cell line. Results show that PTEN may act as a barrier to cancer development by controlling cellular polarity, establishment of cell-cell junctions, paracellular permeability, migration and metastatic potential of human colorectal cancer cells.

## Materials and Methods

### Material and antibodies

The antibodies for detection of PTEN (A2B1), HNF1α (C-19) and HNF4α (C-19) were purchased from Santa Cruz Biotechnology (Santa Cruz, CA). Antibodies raised against E-cadherin and villin were from BD Biosciences (Mississauga, ON, Canada). The antibodies for detection of phospho-AKT (Ser473) and AKT were from Cell Signaling Technology (Danvers, MA). The β-actin antibody was from Chemicon (Temecula, CA). The occludin, claudins and the ZO-1 antibodies were all from Zymed Laboratories (Invitrogen, Burlington, ON, Canada). Monoclonal antibody HSI-14 against sucrase-isomaltase was kindly provided by Dr Andrea Quaroni (Cornell University, Ithaca, NY). CDX2 immunoblotting was performed with a rabbit polyclonal antibody against CDX2 previously characterized [23]. All other materials were obtained from Sigma-Aldrich (Oakville, ON) unless stated otherwise.

### Cell culture

Three colon cancer cell lines were used in the present study:

1-The colorectal adenocarcinoma cell line Caco-2/15 was obtained from A. Quaroni (Cornell University, Ithaca, NY). This cell line provides a unique and well characterized model for the study of gut epithelial differentiation since these cells undergo functional and morphological differentiation to an enterocyte phenotype with microvilli, dome formation, and expression of sucrase-isomaltase several days after reaching confluence [20]–[22]. These cells express truncated *APC* and mutated *p53* but exhibit wild-type *K-Ras*, *β-catenin* and mismatch repair (MMR) proteins; they are therefore microsatellite stable (MSS) [24], [25]. These cells were cultured in Dulbecco's modified Eagle's medium (DMEM, Invitrogen™) containing 10% fetal bovine serum (FBS). 2- The HCT116 cells were obtained from ATCC (CCL-247). These cells grow in multi-layers and are incapable of polarization or intestinal epithelial differentiation [20]. These cells are hMLH1-deficient, express wild-type *p53*, wild-type *APC* and carry activating *K-Ras*, *pi3kca*, and *β-catenin* mutations [26]. These cells are metastatic [27], [28] allowing us to analyse the impact of PTEN loss of expression in advanced stages of colorectal cancer. These cells were cultured in McCoy's medium (Wisent, St-Bruno, Québec, Canada) containing 10% FBS. 3- CT26 cells (kindly provided by Pr Nicole Beauchemin, Université McGill, Canada) were derived from an undifferentiated murine adenocarcinoma which was induced by rectal injection of *N*-nitroso-*N*-methylurethane in Balb/c mice. These cells carry activating *K-Ras* mutations [29], wild-type *p53* and do not express E-cadherin or ZO-1 [30]. CT26 cells were maintained in RPMI medium (Invitrogen™) containing 10% FBS and penicillin-streptomycin.

### Plasmid constructions and lentiviruses production

The lentiviral shRNA expression vector (pLenti6-U6) was constructed as previously described [31]. ShRNA oligonucleotides against human PTEN were designed according to Ambion guidelines (technical bulletin #506) using the siRNA sequences gctaagtgaagatgacaatca (#1), gcacaagaggccctagatttc (#2), gccagctaaaggtgaagatat (#3) with ttcaagaga as the loop sequence. The oligonucleotide-annealed products were subcloned into pLenti6-U6 between the *Bam*HI and *Xho*I sites. An irrelevant pLenti-shGFP (gccacaacgtctatatcatgg) or a mutated pLenti-shPTEN (shMUT) was used as negative control. The mutated pLenti-shPTEN was generated by changing 3 nucleotides in the sequence of the most efficient shRNA against PTEN (#2) resulting in the siRNA sequence gcacaagataacctagatttc. The shRNA against the murine form of Pten (TRCN0000028992) and a corresponding control shRNA (shTGFP) against TurboGFP™ (SHC004) were obtained from Sigma-Aldrich. Lentiviruses produced in 293T cells were used for cell infection according to Invitrogen recommendations (ViraPower Lentiviral Expression System). No induction of *OAS1* gene expression was detected by Q-PCR analysis in the experiments involving lentiviruses infection (data not shown). *OAS1* (2050-oligoadenylate synthetase) is a classic interferon target gene and has been recommended as a key test for interferon induction before attributing a particular response to the gene targeted [32].

### Protein extraction and Western blot analysis

Protein extractions and Western blot analysis were performed as previously described [22].

### Transmission electron microscopy

Samples were processed as previously described [22] and observed on a Hitachi H-7500 transmission electron microscope.

### Scanning electron microscopy

The Caco-2/15 cells were seeded on small lamellae of 12 mm in diameter and fixed as previously described for transmission electron microscopy [22] up to the dehydration step in ethanol. Samples were then critical-point dried with CO_2_ and covered with gold-palladium with a sputter coater. Coated cells were subsequently observed with a JEOL scanning electron microscope (model: JSM-840).

### Determination of transepithelial electric resistance

Caco-2/15 cells were plated on Transwell® permeable membranes (Corning, Acton, MA). TEER were measured 3 and 9 days after the cells had reached confluence with an epithelial Voltommeter (World Precision Instrument, model EVOM-G).

### RNA analysis

Total RNA isolation, RT-PCR and Q-PCRs were performed as previously described [31]. Target expression was quantified relatively to PDGB expression. Primers for each gene were designed at exon-exon junctions using the Primer3 software [33]. Primer sequences and Q-PCR conditions are available on request.

### Migration assays

The assays were performed according to the sharp razor blade technique as previously reported [34].

### Assays of Rac and Cdc42 activities

GTP-bound levels of Rac and Cdc42 were analyzed with a G-LISA activation assay biochemistry kit (Cytoskeleton, Denver, CO) according to the manufacturer's instructions.

### Invasion assays

Invasion assays were performed using BD BioCoat™ Matrigel™ Invasion Chambers with 8-µm polycarbonated filters (BD Biosciences). Cells were seeded in media without serum in the presence of 2 mM hydroxyurea, a pharmacological inhibitor of cellular ribonucleoside reductase to arrest the cell cycle in G1/S phase [35]. Media containing 20% FBS was used as chemoattractant. After an incubation of 48 h at 37°C, non-invasive cells were removed according to the manufacturer's procedure and invading cells were fixed with methanol 100%. Cells were then stained in crystal violet 1%.

### Animal models

Female nude mice CD1 *nu/nu* and Fox Chase SCID Beige mice were purchased from Charles River (Wilmington, MA). All experimental protocols were approved by the Ethics Committee for Animal Experimentation of the Université de Sherbrooke. *Tumor growth*: A total of 2×10^6^ cells suspended in 100 µl DMEM were injected into the dorsal subcutaneous tissue of 5-week-old female mice CD1 *nu/nu*. Mice were sacrificed after 42 days post-injection for Caco-2/15 and 30 days post-injection for HCT116. Tumors were excised and weighed. *Experimental tail vein assays:* The tail-vein of 5-week-old female CD1 *nu/nu* mice or Fox Chase SCID Beige mice was injected with 10^6^ Caco-2/15, HCT116 or CT26 cells suspended in 100 µl DMEM. Animals were sacrificed at any sign of respiratory distress or weight loss, or after 14 days post-injection for CT26, 75 and 35 days post-injection for HCT116 injected respectively in CD1 *nu/nu* and Fox Chase SCID Beige mice and 60 days post-injection for Caco-2/15 cells injected in Fox Chase SCID Beige mice. Lungs were maintained in Bouin's fixative for 24 h. Individual lobes were then separated and the total number of surface-visible metastases was determined.

### Human tumors

Samples of colon cancers and paired normal colon tissues (at least 10 cm from the tumor) have been obtained from patients undergoing surgical resection. Patients did not receive neoadjuvant chemotherapy or radiotherapy. Tissues were obtained after patient's written informed consent, according to the protocol approved by the Institutional Human Subject Review Board of the Centre Hospitalier Universitaire de Sherbrooke. Clinical and pathological informations were obtained from medical records. Adenoma samples were endoscopically unresectable and defined as advanced because of their size larger than 1 cm or by the presence of high-grade dysplasia or villous component. Patient's cancers were histologically classified and graded according to overall TNM staging criteria (based on Tumor-, lymph Node- and Metastatic- status). All tissues were frozen in liquid nitrogen within 15 min from resection as recommended by the Canadian Tumor Repository Network (www.ctrnet.ca). For protein extraction, paired tissues were lysed in Triton sample buffer (100 mM NaCl, 5 mM EDTA [pH 8.0], 50 mM Tris–HCl [pH 7.5], 1% Triton X-100, 5% glycerol, 1 mM PMSF, 0.2 mM orthovanadate, 40 mM β-glycerophosphate, 50 mM NaF, and 2% protease inhibitor cocktail [P 8340, Sigma-Aldrich]) and immunoblotted as previously described [22].

### Data presentation

Typical results shown are representative of 3 independent experiments. Statistics were calculated using Student two tailed t-test. Differences were considered significant at * p≤0.05 or *** p≤0.005. Densitometric analyses were performed using Image J software.

## Results

### PTEN controls colonic epithelial cell polarization and differentiation

**Figure 1:**
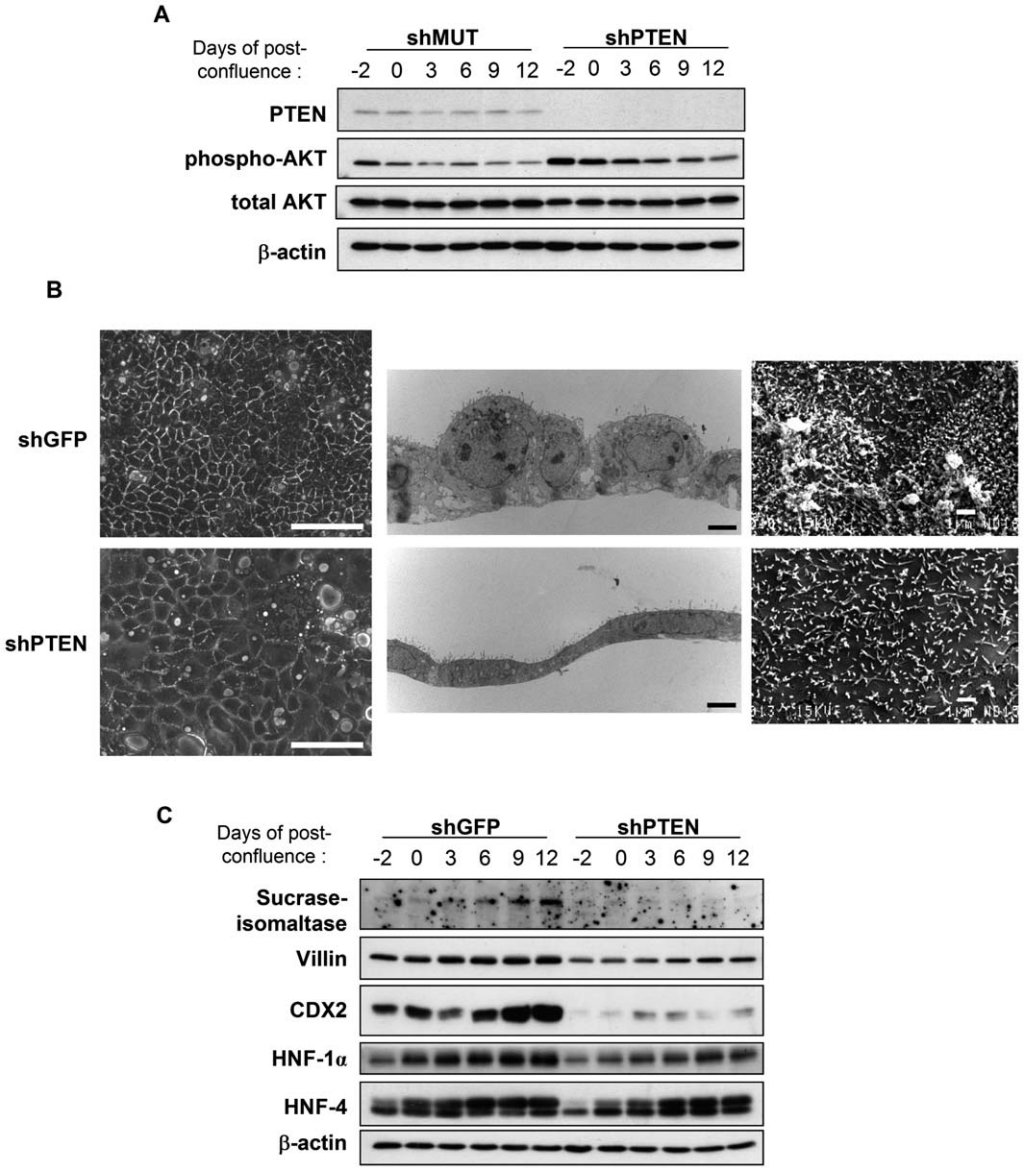
PTEN down-regulation impairs intestinal epithelial cell polarization and differentiation. Caco-2/15 cells were infected with either recombinant lentiviruses encoding a shRNA which specifically knocks-down PTEN (shPTEN) or negative control shRNAs (shGFP or shMUT). **A and C**. After selection of infected cells, Caco-2/15 were lysed at −2, 0, 3, 6, 9 and 12 days post-confluence. Proteins were then analyzed by Western blot. **B**. Left panels: Newly confluent Caco-2/15 cells expressing shGFP or shPTEN were observed by optical microscopy. Scale bars  = 100 µm. Middle panels: Newly confluent Caco-2/15 cells expressing shGFP or shPTEN were fixed and observed by transmission electron microscopy. Scale bars  = 2 µm. Right panels: Newly confluent shGFP or shPTEN expressing-cells were observed by scanning electron microscopy. Scale bars  = 1 µm.

To investigate the impact of PTEN loss of expression on differentiation and polarization of colorectal cancer cells, recombinant lentiviruses encoding short hairpin RNAs (shRNAs) were first developed in order to stably suppress PTEN mRNA levels in Caco-2/15 cells. Several lentiviral constructs were tested for their ability to knock-down PTEN. The most efficient shRNA was designated shPTEN (data not shown). Caco-2/15 cells were henceforth infected with shPTEN lentiviruses or with irrelevant shGFP lentiviruses or lentiviruses expressing a shPTEN with 3 mismatch nucleotides (shMUT) as negative controls. The pLenti6-U6 lentiviral vector, which coexpresses a blasticidin S resistance gene, allowed the selection of pure populations of transduced cells within 7 days. As shown in Figure 1A, PTEN protein levels were almost completely knocked-down in Caco-2/15 cells expressing the shPTEN (>90%); this reduction was maintained during post-confluency. Of note, PTEN down-regulation was also associated with an increase in AKT phosphorylation, the main downstream effector of PI3K, confirming that the PI3K pathway was activated in shPTEN expressing-cells.

To analyze whether PTEN has an impact on Caco-2/15 cell polarization, cell morphology was first examined at different days of confluence. As observed by optical microscopy, cell superficy was significantly increased by 5.3-fold (p<0.005 analyzed by Metamorph software) in newly confluent Caco-2/15 cells expressing shPTEN compared to control cells (Figure 1B, left panels). In addition, transmission electron microscopy analysis showed that newly confluent control cells had already begun to polarize whereas PTEN-deficient cells still maintained a flattened appearance (Figure 1B, middle panels). At 3 days post-confluence, cells with reduced PTEN expression only started to polarize while control cells had already gained their cylindrical shape (data not shown). Down-regulation of PTEN also resulted in a decreased number of microvilli at the cell apical membrane as observed by scanning electron microscopy (Figure 1B, right panels). Of note, expression levels of the functional differentiation markers sucrase-isomaltase and villin were also markedly diminished in PTEN-depleted cells (Figure 1C).

We next verified if PTEN silencing alters the expression of Hepatocyte nuclear factors 1α (HNF1α) and 4 (HNF4α) as well as caudal-related homeobox transcription factor 2 (Cdx2) which are known to control intestinal epithelial cell differentiation [36]. As shown in Figure 1C, while expression levels of HNF4α were modestly reduced in confluent PTEN-deficient Caco-2/15 cells, expression of HNF1α and Cdx2 was markedly decreased (Figure 1C). Taken together, these results indicate that PTEN is important for epithelial cell polarization as well as functional and morphological differentiation of intestinal epithelial cells. Of note, these effects do not seem to be an indirect consequence of alteration in cell proliferation since Caco-2/15 cells expressing shPTEN exhibited the same proliferation rate than control cells and stop to proliferate as soon as they reached confluence (data not shown).

### PTEN down-regulation impairs tight junction integrity in colon epithelial cells

**Figure 2:**
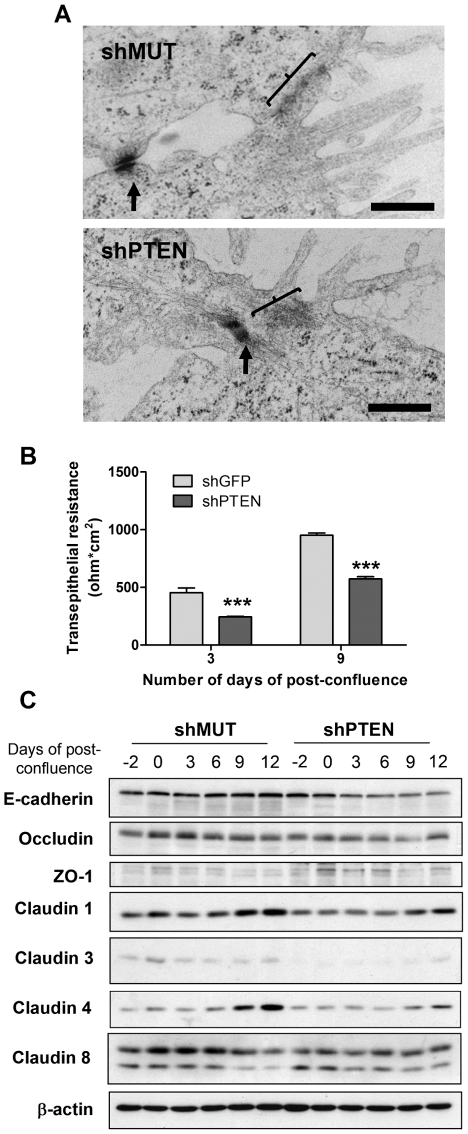
PTEN regulates tight junction integrity and function. **A**. Caco-2/15 cells expressing shMUT or shPTEN were fixed after 3 days post-confluence and observed by transmission electron microscopy. Scale bars  = 500 nm. Arrows indicate adherens junctions while brackets indicate tight junctions. **B**. Caco-2/15 cells were cultivated on porous membranes after which transepithelial resistance was measured in triplicate 3 and 9 days after the cells reached confluence. *** Significantly different at p≤0.005 (Student's t-test). **C**. Caco-2/15 cells expressing shGFP or shPTEN were lysed at -2, 0, 3, 6, 9 and 12 days post-confluence followed by Western blot analysis of junctional proteins.

Since intercellular junctions are regulators of cell polarity [2], [5], we next verified whether the expression of shPTEN had an effect on cell junctions. Under transmission electron microscopy, adherens junctions appeared unaltered (Figure 2A, arrows) in PTEN-deficient Caco-2/15 cells while tight junctions appeared less organized at 3 days post-confluence (Figure 2A, brackets). As a matter of fact, unstructured mass of proteins were systematically observed without tightening of the membrane at the usual position of tight junctions. Accordingly, the barrier function of tight junctions was also compromised as witnessed by the decrease in TEER measured in shPTEN-expressing cells compared to control cells at 3 and 9 days post-confluence (Figure 2B). In order to gain further insight into the manner by which PTEN controls junction integrity, expression levels of several key junctional proteins such as occludin, ZO-1 and claudins [2] were analyzed. Protein expression of claudins 1, 3, 4 and 8 was significantly decreased in both subconfluent and post-confluent shPTEN cells (Figure 2C). Of note, expression of the adherens junction protein E-cadherin was also attenuated in post-confluent shPTEN cells (Figure 2C).

### Loss of PTEN expression in Caco-2/15 cells stimulates migration/invasion, promotes tumor growth but is not sufficient to confer metastatic potential *in vivo*

**Figure 3:**
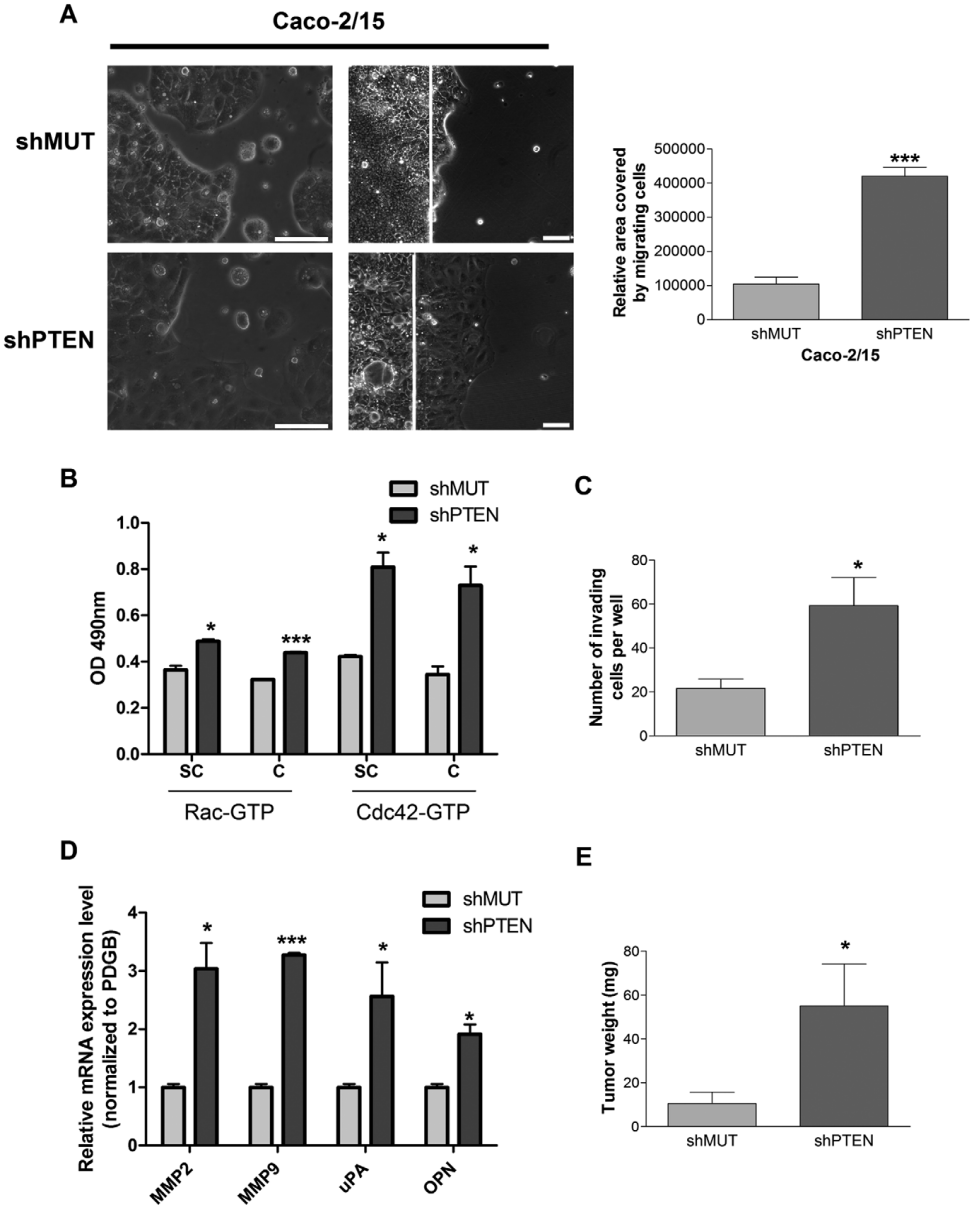
PTEN down-regulation in Caco-2/15 increases migration/invasion capacity and tumorigenic potential. **A**. Left panels: Cell morphology was analyzed by phase contrast microscopy at subconfluence. Right panels: Newly confluent cells stably expressing shMUT or shPTEN were wounded and treated with 2 mM hydroxyurea. After 48 h, movement of the coherent sheet across the linear wound margin (white line) was evaluated by phase contrast microscopy. The graph on the right illustrates the relative area covered by migrating cells as assessed on 5 different wound experiments per condition using Image J software. Scale bars  = 100 µm. **B**. Activated levels of GTP-bound Rac or Cdc42 were analyzed with G-LISA activation assay biochemistry kits on subconfluent (SC) and newly confluent (C) Caco-2/15 cells. **C**. Invasion of cells was studied using Matrigel-coated Transwells. After 48 h, invading cells were fixed, stained with crystal violet 1% and counted. **D**. Subconfluent shMUT and shPTEN-expressing Caco-2/15 cells were lysed and total RNA isolated for gene expression analyzed by Q-PCRs. The relative level of each RNA was calculated using the standard curve method and normalized to the corresponding PDGB RNA level. **E**. 2×10^6^ proliferating Caco-2/15 cells expressing shMUT or shPTEN were injected subcutaneously in 5 nude mice per condition. Tumor weight was evaluated 42 days after injection. * p≤0.05, *** p≤0.005, statistical differences determined using Student's t-test.

Loss of intercellular junctions is well known to be associated with increased cell migration and invasion capacities [4]. Furthermore, PTEN has been shown to control such processes in other cell types [11]. Using the wound-closure assays, the migration of newly confluent control cells was compared with that of shPTEN-expressing populations. As shown in Figure 3A, the ability of shPTEN-expressing Caco-2/15 cells to migrate was markedly increased compared to control cells, as determined by measuring the relative area covered by migrating cells. We also observed that down-regulation of PTEN markedly stimulated Caco-2/15 cell detachment after trypsinization (data not shown). These data indicate that PTEN gene transcript silencing in CRC cells enhances their capacity to spread and migrate. Since PTEN has been shown to influence migration through the activation of the small GTPases Rac1 and Cdc42 [37], we next analyzed whether their respective expression and activity was affected by loss of PTEN expression. As shown in Figure 3B, both Rac and Cdc42 displayed higher GTP-bound levels in subconfluent and confluent shPTEN-expressing cells compared with control cells.

The effect of PTEN deficiency on invasion was also determined using BD Biocoat Matrigel invasion chambers. As shown in Figure 3C, Caco-2/15 cells expressing the shPTEN clearly exhibited increased invasive capacities in comparison to shMUT-expressing cells. Invasion not only involves breakdown of cell-cell junctions and increased motility of tumor cells but also focal proteolysis of the extracellular matrix. As shown in Figure 3D, Q-PCR analyses revealed that expression levels of matrix metalloproteinases 2 and 9 (MMP2 and MMP9), osteopontin (OPN) and uPA (urokinase-type plasminogen activator) were significantly up-regulated in shPTEN-expressing Caco-2/15 cells.

The tumorigenicity of Caco-2/15 cell populations was next assessed following subcutaneous injection into the flank of nude mice. As shown in Figure 3E, down-regulation of PTEN expression in Caco-2/15 cells severely increased their capacity to induce tumors in vivo.

Finally, we investigated whether PTEN reduction in Caco-2/15 cells is sufficient to induce tumor metastasis *in vivo*, through the use of experimental metastasis tail vein assay. Fox Chase SCID Beige mice injected with control or PTEN-deficient Caco-2/15 cells into the tail vein failed to develop metastases even at 60 days after injection (data not shown), indicating that PTEN downregulation is not sufficient to confer a metastatic potential to well-differentiated CRC cells.

### Loss of PTEN expression stimulates migration/invasion, enhances tumor growth and induces metastatic potential of HCT116 cells *in vivo*

**Figure 4:**
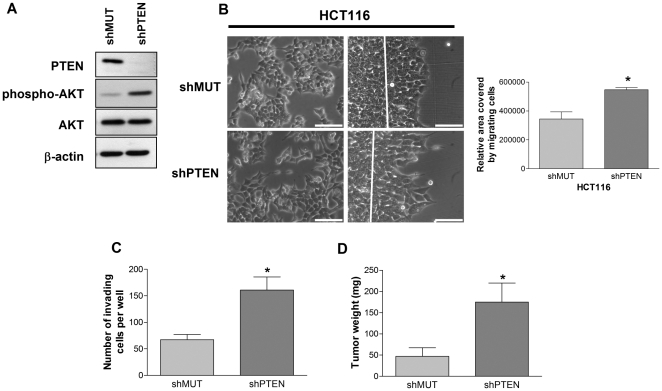
PTEN down-regulation in HCT116 increases migration/invasion capacity and tumorigenic potential. **A**. HCT116 cells were infected either with recombinant lentiviruses encoding shMUT or shPTEN. After selection of infected cells, proliferating cells were lysed and protein expression was analyzed by Western blot. **B**. Left panels: Cell morphology was analyzed by phase contrast microscopy at subconfluence. Right panels: Newly confluent cells were wounded and treated with 2 mM hydroxyurea. After 48 h, movement of the coherent sheet across the linear wound margin (white line) was evaluated by phase contrast microscopy. The graph on the right illustrates the relative area covered by migrating cells as evaluated on 5 different wound experiments per condition using Image J software. Scale bars  = 100 µm. **C**. Invasion of cells was studied using Matrigel-coated Transwells. After 48 h, invading cells were fixed, stained with crystal violet 1% and counted. **D**. 2×10^6^ proliferating HCT116 cells expressing shMUT or shPTEN were injected subcutaneously in 5 nude mice per condition. Tumor weight was evaluated 30 days after injection. * Significantly different at p≤0.05.

The impact of PTEN silencing was also assessed in the microsatellite-unstable dedifferentiated cell line HCT116. In these cells, PTEN silencing also resulted in enhanced phospho-Akt (Figure 4A), enhanced capacity of both migration upon wounding (Figure 4B) and invasion (Figure 4C), in addition to being associated with a significant increase in Rac activity (1.5-fold, p<0.005) and OPN expression (1.9-fold, p<0.005) (data not shown). Of note, expression levels of MMP-2, MMP-9 and uPA in control HCT116 cells were already markedly elevated and PTEN silencing did not further enhance mRNA levels of these molecules (data not shown). The tumorigenicity of HCT116 cell populations was also assessed following subcutaneous injection into the flank of nude mice. As shown in Figure 4D, down-regulation of PTEN expression in HCT116 cells increased their capacity to induce tumors *in vivo*.

**Figure 5:**
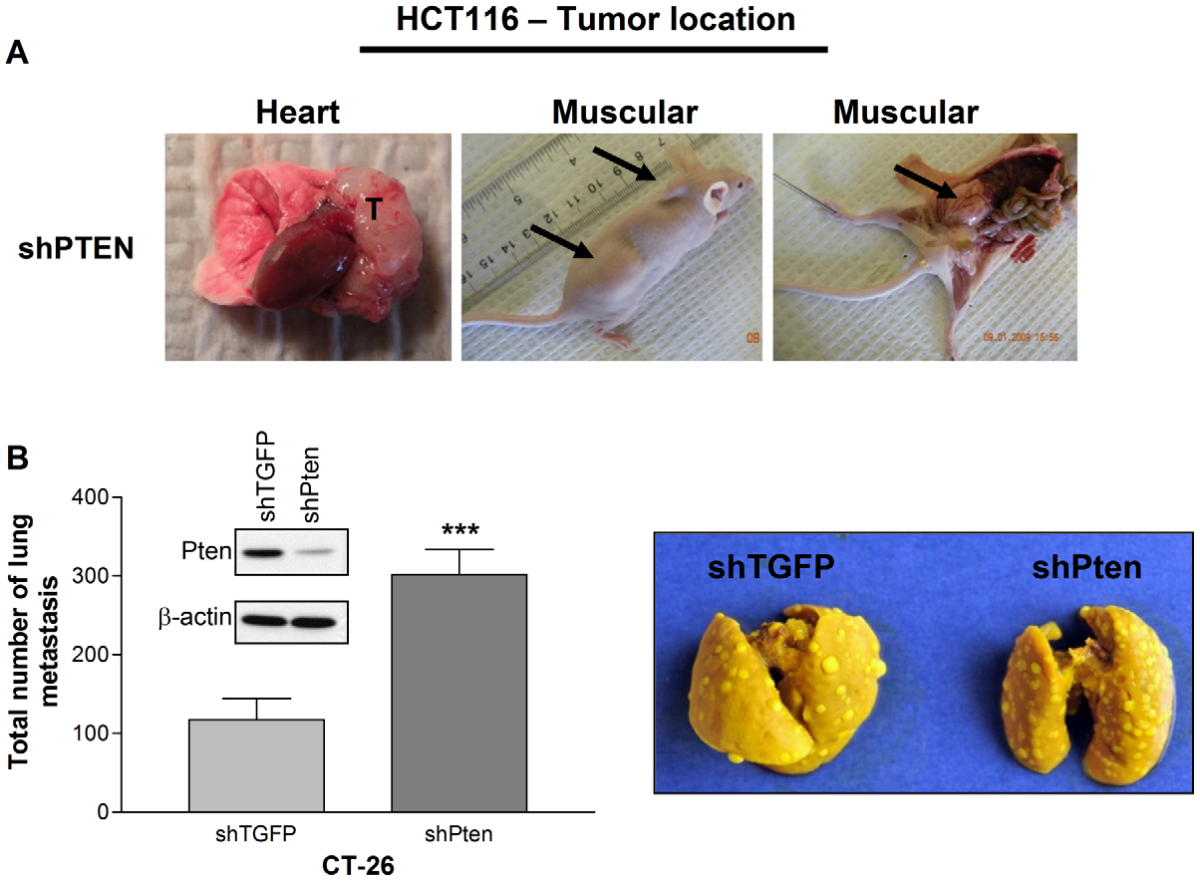
PTEN down-regulation increases metastatic potential of HCT116 and CT26 cells *in vivo*. **A**. Proliferating HCT116 cells expressing shMUT or shPTEN were injected in the lateral tail-vein of 12 mice per condition. Mice injected with HCT116-expressing shPTEN developed tumors in (various, multiple) locations such as the heart and dorsal muscle 50–75 days after tail-vein injection. T: tumor. **B**. CT26 cells were infected either with recombinant lentiviruses encoding a shRNA which specifically knocked-down the murine form of PTEN (shPten) or with a negative control shRNA (shTGFP). After selection of infected cells, proliferating cells were lysed and protein expression was analyzed by Western blot. Proliferating CT26 cells expressing shTGFP or shPten were also injected in the lateral tail-vein of 6 mice per condition. Total number of detectable pulmonary metastasis was evaluated 14 days after tail-vein injection. Significantly different at *** p≤0.005.

Finally, we investigated whether PTEN reduction in HCT116 cells is sufficient to induce tumor metastasis *in vivo*, through the use of tail vein assay. After 75 days post-injection, none of the mice injected with control cells exhibited tumors at necropsy, while 25% of the mice having received cells expressing shRNA against PTEN developed tumors in various locations such as the heart and dorsal muscles (Figure 5A). This pattern of “multi-location” metastases has previously been reported with HCT116 clones expressing *pi3kca* mutant [38]. In addition, preliminary results obtained with Fox Chase SCID Beige mice, which are more succeptible to metastases development, suggest that injection of PTEN-deficient cells in these mice results in much more pulmonary and “multi-location” metastases than injection of control HCT116 cells (data not shown). Experiments were also conducted using the mouse metastatic colon carcinoma cell line CT26 in order to verify whether PTEN silencing also enhanced their capacity to develop lung metastasis in allogeneic mice. Reduction of PTEN expression (Figure 5B) in these cells also resulted in enhanced expression levels of phosphorylated Akt (not shown). Importantly, PTEN silencing in CT26 cells significantly increased their capacity to develop metastases in the lung (>3-fold) after their injection into the tail vein within 14 days (Figure 5B). Thus, these data suggest that PTEN downregulation does enhance the metastatic potential of CRC cells which have already acquired metastatic properties.

### PTEN protein expression is frequently diminished in colorectal cancer patients

**Figure 6:**
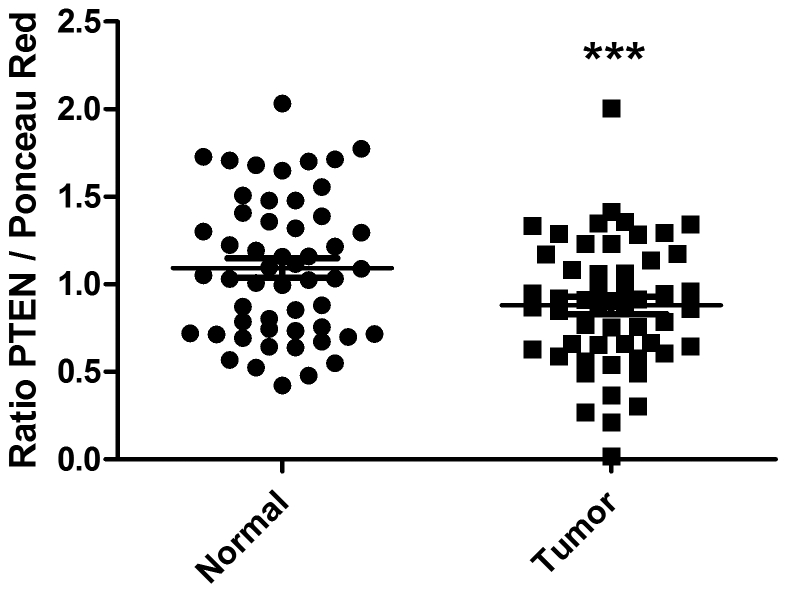
PTEN expression is decreased in colorectal cancers. PTEN protein expression was investigated by Western blot in 53 paired samples of colon cancers (resection margins and primary tumors). Expression levels were normalized to the intensity of Ponceau red staining and to a reference sample, resulting in a dimensionless value (arbitrary units-AU). Amounts of PTEN protein in tumor tissues relative to their matched normal samples were analyzed by paired t-test. *** Significantly different at p≤0.005.

Although *PTEN* gene mutations are rare [13], [16], [39], [40], several studies have reported, by using immunohistochemical detection methods, a decrease of PTEN expression in colorectal cancer tissues [14]–[17], [41]. However, some variability in PTEN immunostaining has been observed, with studies reporting either nuclear or cytoplasmic PTEN localization. Additionally, it has been reported that some antibodies frequently used gave a non-specific PTEN signal on paraffin-embedded tissues [42]. Therefore, PTEN protein expression was investigated by Western blot analysis in 53 paired samples of colon cancer (resection margins and primary tumors). As observed in Figure 6, relative amounts of PTEN protein were effectively found to be significantly reduced in these colorectal tumors compared to corresponding normal specimens (p≤0.005, paired t-test). In fact, expression of PTEN protein was found to be significantly reduced by 40% in 33/53 tumors (*P*<0.0001, paired *t*-test), regardless of tumor stage and grade (5 to 16 biopsies per stage).

## Discussion

Tissue transformation in cancer is frequently associated with loss of cell and tissue polarity, although the molecular mechanisms underlying this loss are not known. It is likely that the transformation process involves alteration in polarity gene expression and/or their subcellular protein localization resulting in functional inactivation of polarity pathways [7]. In this respect, PTEN has recently emerged as a key determinant of epithelial polarity [9]. Indeed, recent analyses *in vivo*, in primary cells or using cultured cells retaining significant cellular architecture and polarity, have shown that PTEN is enriched at specialized sites, in particular, at the apical domains of polarized epithelial cells. This epithelial polarization of PTEN was initially demonstrated in *Drosophila* [43], [44] and subsequently in the chick embryo epiblast epithelium [45] as well as in polarized T84 and Caco-2 colon cells and MDCK kidney cells in 3D culture [46]. Consequently, the substrate of PTEN, PtdIns(3,4,5)P_3_, is stably localized at the basolateral membrane and is excluded from the apical plasma membrane of epithelial cells [47], [48]. Of note, PtdIns(3,4,5)P_3_ has been reported to act as a key determinant of the epithelial basolateral surface in mammalian cells [49]. Addition of exogenous PtdIns(3,4,5)P_3_ to the apical surface of filter-grown MDCK cells causes the relocalization of basolateral proteins to the apical surface and the removal of apical proteins from the apical surface [49]. Conversely, growth of MDCK or Caco-2/15 cells in the presence of low concentrations of inhibitors of PI3K which convert PtdIns(4,5)P_2_ to PtdIns(3,4,5)P_3_, causes the size of the lateral membrane and the height of the cells to be diminished [22]. Therefore, by controlling the levels of PtdIns(4,5)P_2_ and PtdIns(3,4,5)P_3_, PI3K and PTEN thus stringently control the signaling from phosphoinositides defining epithelial plasma membrane identity and epithelial polarity.

There is clear genetic evidence for a central role for both of these enzymes in tumorigenesis: somatic mutations that target PTEN and the α catalytic subunit of PI3K (pi3kca) are frequent occurrences in human cancer, resulting in increased activity of the PtdIns(3,4,5)P_3_ signaling pathway [50]. In colorectal cancer specifically, the *pik3ca* gene is mutated in 20–25% of CRCs while *pik3ca* mutations occurring in the hotspots located in exon 9 and exon 20 are oncogenic in CRC cell models [51]. Of note, *pik3ca* gene abnormalities appear to occur at relatively late stages of neoplasia, near the time where tumors begin to invade and metastasize [38]. *PTEN* mutations are rare in colorectal cancers [13], [16], [39], [40] although infrequent *PTEN* mutations in mononucleotide repeat sequences have been reported in sporadic colon cancers, mainly in association with microsatellite instability [52]. Herein, we observed that PTEN protein expression was decreased in CRCs in approximately 60% of studied patients, in accordance with several report based on immunohistochemical detection tools [14]–[16]. This suggests that epigenetic mechanisms of *PTEN* downregulation are frequent in CRCs [13]. Although this relationship remains to be elucidated in prospective studies, reduction of PTEN may be important for the adenoma-colon cancer sequence.

Herein, through a RNA interference approach, we analyzed the role of PTEN in the poorly tumorigenic well-differentiated cell line Caco-2/15, derived from a typical colon adenocarcinoma. Our data demonstrate that reduction of PTEN expression in these cells severely impaired their functional polarity which was associated with reduced levels of tight junction proteins and decreased transepithelial resistance. In addition, Caco-2/15 cells containing a low level of PTEN had an enhanced capacity to migrate and invade *in vitro* and to form tumors *in vivo* in nude mice. However, while PTEN silencing was able to further enhance the metastatic potential of “de-differentiated” colorectal cancer cells (HCT116 and CT26), it was not sufficient to confer metastatic properties to Caco-2/15 cells *in vivo*.

PTEN has previously been shown to interact with cell adhesion complexes and to stabilize adherens junctions and focal adhesions thereby allowing a reduction of invasiveness in a range of cancer cells [53]–[56]. We show herein that loss of PTEN also impairs the expression of proteins associated with tight junctions, namely claudins-1, -3, -4 and -8. Claudins served as the backbone of tight junctions by forming continuous networks of intramembranous fibrils (tight junction strands) [2]. This change in expression of claudins in shPTEN-expressing Caco-2/15 cells was also accompanied by a decrease in transepithelial resistance in confluent monolayers of these cells indicating that PTEN plays a role in the regulation of intestinal barrier function. However, because claudins represent a family of more than 20 different four-transmembrane proteins, we cannot exclude that other claudins are also modulated by PTEN silencing and contribute to the observed effect on transepithelial resistance. The molecular mechanisms by which PTEN affects the expression of these claudins, and consequently paracellular permeability, are yet unknown. Previous studies have demonstrated that PTEN enhances the expression of Cdx2 [57], [58], a transcription factor known to positively regulate gene expression of claudin-2 [59], claudin-3 and claudin-4 in intestinal epithelial cells [60]. Our data indicate that Cdx2 as well as HNF1α and HNF4α are significantly downregulated following PTEN silencing in Caco-2/15. Moreover, HNF4α has been shown to trigger expression of occludin, claudin-6, claudin-7 in mouse F9 cells [61] and claudin-15 in mouse intestinal epithelium [62]. More recently, knockdown of HNF4α in Caco-2 parental cells decreased the mRNA and protein levels of claudin-1, occludin and ZO-1 in association with disruption of the epithelial barrier function [63]. Thus, while further experiments are needed to identify the exact molecular mechanisms involved in this process, one could speculate that PTEN controls intestinal barrier function by altering Cdx2 and HNF4α expression and consequently, expression of tight junction proteins. In this regard, PTEN activity has recently been shown to induce an intestinal differentiation of gastric carcinomas by increasing CDX2, claudin-3 and claudin-4 expression [57], hence supporting this hypothesis.

Despite accumulating evidences showing a positive link between low PTEN expression and intestinal transformation and cancer, the specific involvement of loss of PTEN signaling in polarization, tumorigenesis and metastasis of colonic cells remains elusive. Colon cancer cells which contain mutant *pi3kca* and therefore enhanced PtdIns(3,4,5)P_3_ levels possess a significant advantage in cell growth and invasion [38]. Accordingly, the present results show that PTEN silencing in Caco-2/15 and HCT116 colorectal cancer cells significantly enhanced their capacity to form tumors *in vivo*. We also found that reduction of PTEN expression in these CRC cell lines induced their migration capacity *in vitro* probably by promoting the activation of Rac and Cdc42. Indeed, although many of the effects of PTEN on cell growth and survival are believed to be mediated through its inhibition of PtdInsP3-dependent protein kinase Akt [12], the effects of PTEN on cell polarity and migration appear to be rather mediated through other PtdIns(3,4,5)P_3_-dependent pathways, in particular, the small GTPases of the Rac and Cdc42 families [37], [45], [46], [64], [65]. In the present study, results revealed that PTEN silencing conferred CRC cells with an enhanced invasive capacity to migrate *in vitro* probably by inducing the expression of MMP-2, MMP-9 and uPA as it has already been observed in prostate cancer cells [66]. These effects of PTEN silencing are likely mediated by enhanced Akt activity since downstream activation of Akt-dependent signaling pathways has previously been shown to promote expression of angiogenic and metastatic genes including *uPA*, *COX2*, *MMP-2*, *MMP-9* and *osteopontin* in CRC cell lines [67], [68].

Loss of cell-cell adhesion followed by the dissociation of epithelial structures is generally referred to as epithelial-mesenchymal transition (EMT). This phenotypic switch plays a fundamental role in morphogenetic tissue remodeling during embryogenesis. However, inappropriate reactivation of the EMT program is commonly observed during transition of benign adenomas to metastatic carcinomas [5]. Recently, Bowen et al. [19] reported that PTEN knockdown increases migration and invasion of the CRC cell lines HCT116 and SW480 which is associated with change in E-cadherin expression, leading the authors to conclude that PTEN loss induces cellular changes consistent with EMT. Unfortunately, the expression of Snail1 and Snail2 as well as the expression of other key markers of EMT such as vimentin and N-cadherin were not analyzed in their study. Furthermore, HCT116 and SW480 cell lines are not the most accurate models to study EMT because they are not differentiated, not polarized and do not exhibit typical and functional apical junctional complexes. Data obtained in the present study suggest that the downregulation of PTEN is not sufficient to trigger EMT-like phenotype and metastatic properties in colorectal adenocarcinoma cells retaining significant cellular epithelial architecture and polarity such as Caco-2/15 cells. Indeed, although PTEN downregulation resulted in severe reduction of polarity associated with a decrease in tight junction proteins, expression of mesenchymal markers such as N-cadherin and vimentin was not increased as confirmed by Western blot analysis (data not shown). In addition, phase-contrast and electron microscopy analyses demonstrated that Caco-2/15 cells containing a low level of PTEN failed to acquire a fibroblastic or scattered morphology and retained their capacity to form some cell-cell contacts. More importantly, PTEN-deficient Caco-2/15 cells were not able to form metastases when injected into the tail vein. This suggests that PTEN silencing during CRC development may not be sufficient for a complete loss of epithelial cell polarity and for cell detachment. Interestingly, we [69] and others [70] have recently reported that the conditional deletion of Pten in the mouse intestinal epithelium was not sufficient to initiate tumor formation. However, loss of Pten in the context of APC deficiency can lead to the development of adenocarcinoma [70]. Therefore, additional abnormalities must co-exist in addition to the loss of PTEN for colon tumor cells to become fully invasive and metastatic. Such phenomenon has previously been reported in the prostate where loss of epithelial PTEN function leads to invasive lesions or even metastasis only in combination with *KRAS* and *SMAD4* mutations [71]. Evidences from the literature suggest that KRAS may in fact be a plausible candidate in CRC. Indeed, *KRAS* is mutated in nearly 50% of tumors at a relatively early stage of the carcinogenic process [72]. Furthermore, recent studies indicate that KRAS signaling contributes to metastasis formation in CRC [73]–[75]. Of note, Caco-2 cells do not carry mutations in either *KRAS* or *BRAF* genes [25] whereas CT26 and HCT116 cells exhibit an activating mutation in codons 12 and 13 respectively of the *KRAS* proto-oncogene [26], [29]. Inhibition of the KRAS downstream effectors, MEK1/2, in CRC cell lines carrying activating *KRAS* mutations (HCT116, DLD1, LoVo, SW480) has also been shown to inhibit expression of Snail1 and Snail2 [75], indicating that expression of these key EMT factors is likely dependent on KRAS signaling in these cell lines. Snail1 and Snail2 transcription factors are the major proteins implicated in repression of E-cadherin, which is the key molecular change occurring during EMT [5]. Moreover, the reduction and loss of E-cadherin expression has been reported in advanced colorectal carcinomas [6] and has been considered to augment cellular dissemination and tumor metastasis [76]. Of particular interest, experiments conducted to silence the expression of E-cadherin not only showed a morphological shift from an epithelial to a fibroblastoid phenotype, characteristic of EMT, but also a concomitant increase in invasive cell behavior [5]. In keeping with this observation, we (data not shown) and others [30] demonstrated that CT26 cells do not express E-cadherin while HCT116 cells express considerably less E-cadherin in comparison to Caco-2/15 cells. Hence, loss of E-cadherin also appears to be a prerequisite for tumor progression and not just a consequence of tumor dedifferentiation.

In summary, the present studies performed in colorectal cancer cells demonstrate that the expression level of PTEN may have a profound influence on the susceptibility of tumor cells to polarize, migrate, invade and metastasize. However, our results also suggest that reduction of PTEN during the development of intestinal epithelial-derived tumors is not sufficient by itself to induce EMT and metastasis. The large number of processes affected by PTEN downregulation could explain the association between low PTEN expression and poor prognosis in colorectal cancer patients [16], [41], [77], although we and others did not find any significant relation between PTEN expression and grade or stage of colorectal cancers (our results and [15]). We could not exclude however that the phosphatase activity of PTEN might be further inhibited by other mechanisms such as PTEN phosphorylation, ubiquitination, acetylation or oxidation [9] in late stages of carcinogenesis. This strengthens the need to understand the cellular mechanisms by which PTEN phosphatase activity is controlled. Together with the accumulating evidence that loss of cellular polarity and tissue architecture can be the driving force in tumor progression rather than its by-product [78], the current data indicate that studies pertaining to PTEN in epithelial biology may be highly relevant to the tumor suppressor functions of PTEN.
